# Draft Genome Sequence of *Mycobacterium boenickei* CIP 107829

**DOI:** 10.1128/genomeA.00292-17

**Published:** 2017-05-04

**Authors:** Amar Bouam, Catherine Robert, Olivier Croce, Anthony Levasseur, Michel Drancourt

**Affiliations:** Aix Marseille Univ, URMITE, UM63, CNRS 7278, IRD 198, INSERM 1095, IHU Méditerranée Infection, Marseille, France

## Abstract

*Mycobacterium boenickei* is a rapidly growing mycobacterium isolated for the first time from a leg wound in the United States. Its 6,506,908-bp draft genome exhibits a 66.77% G+C content, 6,279 protein-coding genes, and 59 predicted RNA genes. *In silico* DNA-DNA hybridization confirms its assignment to the *Mycobacterium fortuitum* complex.

## GENOME ANNOUNCEMENT

*Mycobacterium boenickei* has been characterized as one of the members of the former *M. fortuitum* third biovariant complex sorbitol-negative (1). It is an acid-fast, Gram-positive, pleomorphic bacterium, forming white to slightly beige colonies in less than 7 days. It was initially isolated from a leg wound in the United States (1). *M. boenickei* encodes the intrinsic macrolide resistance gene *erm* (2), rendering it resistant to clarithromycin (3).

We performed the whole-genome sequencing of *M. boenickei* in order to describe its genomic content and to determine its phylogenetic relationships for facilitating the detection and identification of this species.

*M. boenickei* CIP 107829 (and Collection de Souches de l’Unité des Rickettsies, CSUR 25) was cultured in MGIT Middlebrook liquid culture (Becton Dickinson, Le Pont-de-Claix, France) at 37°C. *M. boenickei* CIP 107829 genomic DNA was sequenced by Illumina MiSeq runs (Illumina Inc, San Diego, CA, USA) with the mate-pair strategy using the Nextera mate-pair sample prep kit (Illumina). The index representation for *M. boenickei* CIP 107829 was determined to be 8.67%. A total of 1,391,382 paired-end reads were filtered per the read qualities. These reads were trimmed using Trimmomatic (4) and then assembled into scaffolds using SPAdes version 3.5 (5, 6) before manual finishing. SSPACE version 2 (7) and Opera version 2 (8) were used to combine the contigs, and GapFiller version 1.10 (9) was used to close gaps in the scaffolds. This yielded a draft genome comprising 14 scaffolds composed of 14 contigs, for a total of 6,506,908 bp with a 66.77% G+C content. Noncoding genes and miscellaneous features were predicted using RNAmmer (10), ARAGORN (11), Rfam (12), Pfam (13), and Infernal (14). Coding DNA sequences were predicted using Prodigal (15), and functional annotation was achieved using BLASTp against the GenBank database (16) and the Clusters of Orthologous Groups (COGs) database (17, 18). The genome was shown to encode 59 predicted RNAs, including 3 complete ribosomal operons (5S rRNA, 16S rRNA, and 23S rRNA genes) and 50 tRNAs. A total of 4,828 genes (76.89%) were assigned a putative function, 116 genes (1.85%) were identified as ORFans (open reading frames with no matches in the databases), and the remaining 1.035 genes (16.48%) were annotated as hypothetical proteins. The *M. boenickei* CIP 107829 genome was further incorporated into *in silico* DNA-DNA hybridization (DDH) (19) with reference genomes selected based on 16S rRNA gene proximity; DDH values were estimated using the GGDC version 2.0 online tool (20). This analysis yielded 36.35% ± 3.46 with *M. septicum* DSM 44393, 31.55% ± 3.46 with *M. fortuitum* strain CT6, 21.55% ± 3.32 with *M. cosmeticum* strain DSM 44829, 20.95% ± 3.32 with *M. neoaurum* VKM, 20% ± 3.25 with *M. abscessus* subsp. *bolletii* strain MA 1948, and 19.9% ± 3.25 with *M. chelonae* CCUG 47445, confirming at the genome level the taxonomic assignment of *M. boenickei* to the *M. fortuitum* complex.

### Accession number(s).

The *M. boenickei* CIP 107829 genome sequence has been deposited at EMBL under the accession number FUWC00000000.
